# Using Win-Win Strategies to Implement Health in All Policies: A Cross-Case Analysis

**DOI:** 10.1371/journal.pone.0147003

**Published:** 2016-02-04

**Authors:** Agnes Molnar, Emilie Renahy, Patricia O’Campo, Carles Muntaner, Alix Freiler, Ketan Shankardass

**Affiliations:** 1 Centre for Research on Inner City Health, Li Ka Shing Knowledge Institute, Toronto, Ontario, Canada; 2 Dalla Lana School of Public Health, University of Toronto, Toronto, Ontario, Canada; 3 Bloomberg School of Nursing, University of Toronto, Toronto, Canada; 4 Department of Health Sciences, Wilfrid Laurier University, Waterloo, Ontario, Canada; Penn State College of Medicine, UNITED STATES

## Abstract

**Background:**

In spite of increasing research into intersections of public policy and health, little evidence shows how policy processes impact the implementation of Health in All Policies (HiAP) initiatives. Our research sought to understand how and why strategies for engaging partners from diverse policy sectors in the implementation of HiAP succeed or fail in order to uncover the underlying social mechanisms contributing to sustainable implementation of HiAP.

**Methods:**

In this explanatory multiple case study, we analyzed grey and peer-review literature and key informant interviews to identify mechanisms leading to implementation successes and failures in relation to different strategies for engagement across three case studies (Sweden, Quebec and South Australia), after accounting for the role of different contextual conditions.

**Findings:**

Our results yielded no support for the use of *awareness-raising* or *directive strategies* as standalone approaches for engaging partners to implement HiAP. However, we found strong evidence that mechanisms related to *“win-win” strategies* facilitated implementation by increasing perceived *acceptability* (or buy-in) and *feasibility* of HiAP implementation across sectors. Win-win strategies were facilitated by mechanisms related to several activities, including: the development of a shared language to facilitate communication between actors from different sectors; integrating health into other policy agendas (eg., sustainability) and use of dual outcomes to appeal to the interests of diverse policy sectors; use of scientific evidence to demonstrate the effectiveness of HiAP; and using health impact assessment to make policy coordination for public health outcomes more feasible and to give credibility to policies being developed by diverse policy sectors.

**Conclusion:**

Our findings enrich theoretical understanding in an under-unexplored area of intersectoral action. They also provide policy makers with examples of HiAP across wealthy welfare regimes, and improve understanding of successful HiAP implementation practices, including the win-win approach.

## Introduction

Health in All Policies (HiAP) has been defined as a policy strategy for improving population health and supporting health equity. The approach involves coordinating policy sectors to address the social determinants of health that comprise root causes of social and health inequalities. In practice, HiAP initiatives apply governance strategies, structures and tools mainly outside of the health sector and at all levels of government for implementation [1].

In spite of increasing awareness about the benefits of HiAP approaches [2,3], and the uptake of HiAP in many settings globally [4], few attempts have been made to synthesize evidence of how and why specific strategies, structures and tools have (and have not) worked to facilitate HiAP implementation [2,5–7]. While there is a growing discourse on explaining the implementation of intersectoral action more broadly (e.g., [8,9]), systematic research methods to pinpoint the social mechanisms that regularly enable or hinder the implementation of HiAP have not been applied.

We have previously argued the central importance of fostering acceptability and feasibility of HiAP implementation across governments in order to foster sustainable initiatives [10]. This study provides evidence on the social mechanisms for how and why certain governance strategies are effective at agenda setting and capacity building to get buy-in from policymakers across sectors and non-governmental partners for HiAP initiatives, and what specific resources are needed for sustaining buy-in. We apply an explanatory, multiple-case study design informed by a scientific realist perspective [11] to understand what mechanisms constituted facilitators and barriers to the implementation of HiAP in Quebec, Sweden and South Australia.

## Conceptual Framework

**Fig 1:**
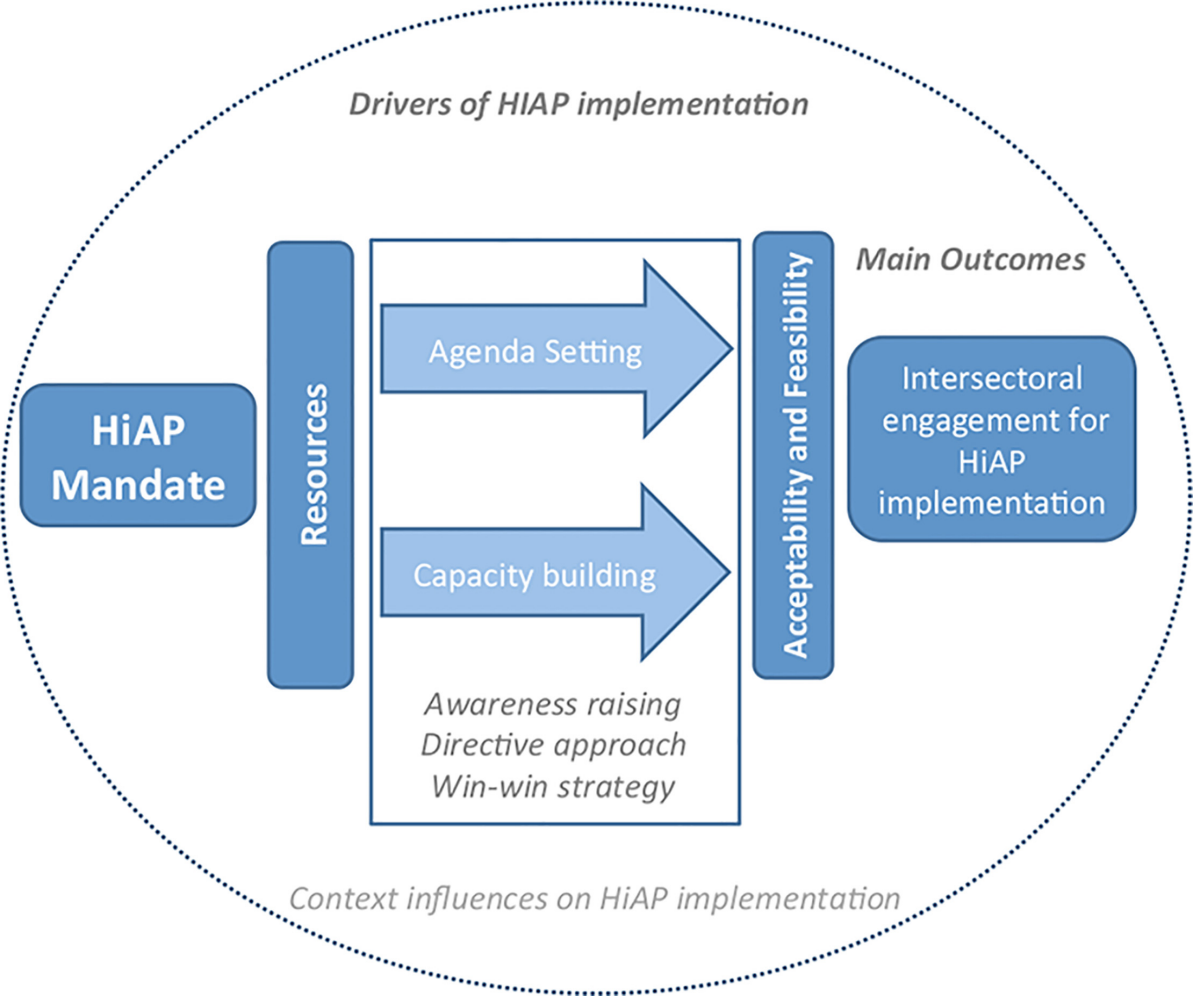
Conceptual Framework for Implementation of HiAP.

As described in Fig 1, we focus on understanding how specific mandates for HiAP and the use of certain resources for implementation were utilized to facilitate processes of agenda setting (ie., a key process for implementing HiAP requiring the alignment of multiple interests to facilitate ‘buy-in’ by potential collaborators) and capacity building (ie., a key process for implementing HiAP contingent on the presence of appropriate and/or adequate human, information, financial or infrastructural resources for implementation of a HiAP strategy) critical for HiAP implementation [10].

Here, we focused on testing the importance of three related governance strategies (our three main hypotheses) that had been discussed in the literature at the time of our study (eg., [1, 12], as well as by our team [13]. These included *awareness raising*, *directive*, and *win-win* strategies for achieving buy-in for HiAP from governmental and non-governmental partners (Fig 1). In brief, *awareness raising* refers to the process of articulating to policymakers why intersectoral action is needed to address population health and equity with the expectation that they will buy in and participate ([13]; see also [1,14]). A *directive* approach involves convincing potential partners to adopt policies and measures that directly support health objectives, with consideration of sector-specific interests being less important. Finally, the *win–win* strategy aims for health gains without diminishing the primary intention of participating sectors or agencies enabling the prioritization of social and economic outcomes [12].

In order to maintain intersectoral engagement across diverse jurisdictions in the implementation of HiAP, we have previously argued that both the acceptability (ie., sectors buy in to policy coordination for health and equity) and feasibility (ie., sectors have the capacity to effectively collaborate on health and equity) are important outcomes to consider. This framework also predicts that contextual differences about the implementation of HiAP in each setting will help to explain differences in the salience of these strategies in each case. For example, we have previously argued the importance of having prior experience with intersectoral action (ISA) and health, which could influence whether stakeholders see value in HiAP and know how to use the governance tools being introduced for HiAP.

## Methods

### Explanatory multiple case study method

We used a multiple explanatory case study approach [15,16]–including a scientific realist perspective [10]–to test hypotheses about what drives HiAP implementation in specific national and high-level sub-national jurisdictions, eg., states and provinces; ie., not solely at the municipal level. This approach falls within realist methods (eg., [17]), which are informed by realist philosophy [18] in that they assume that interventions are objective and can be known via uncovering mechanisms. We focus on “social” mechanism, which are the interactive, oft-hidden processes that cause the implementation of HiAP. Social mechanisms (hereafter, mechanisms) involve at least two persons engaged in a political, cultural or economic relation [19,20].

Our case study methodology has been described elsewhere [10], but we include a summary of steps for conducting single case studies and cross-case analysis in Table 1. In brief, this approach encompasses two parts: (1) Single explanatory case studies, where we focus on learning intensively about causal mechanisms that explain progress (and lack thereof) in the implementation of HiAP; (2) Cross-case analysis, where we test our study hypotheses to develop a theoretical understanding of how implementations works across case settings.

**Table 1:** Activities associated with each step of the case study process.

3Step in Case Study Process	Activities	Description
**Create initial theory and hypotheses about how agenda setting and capacity building sustain HiAP**	Consult literature	Conduct selective review of grey and peer review literature for theories about sustaining HiAP initiatives
	Engage stakeholders and knowledge users	Consult with stakeholders and knowledge users about their experiences with HiAP and priorities for evidence needs
	Develop and prioritize hypotheses	Draw from literature and stakeholder engagement activities to develop cross-case hypotheses with a focus on knowledge user—priority issues
	Select cases	Select cases based on the i) period of initiation,^1^ ii) richness of literature iii) similarity and difference between cases
**Collect and synthesize data within each case & generate single case study reports**	Consult grey and peer-review literature	Collect literature on HiAP for each case that is relevant for the testing of hypotheses by undertaking a systematic search for grey and peer-review literature that is relevant for the testing of hypotheses.^2^
	Conduct key informant interviews with HiAP experts	Identify HiAP experts with strong experience working on HiAP by undertaking a search for prominent authors of reports on the case as well as through snowball sampling. Confirm expertise and experience through screening potential interviewees. Inquire about evidence related to the hypotheses by using a semi-structured interview guide, and transcribing the interview for systematic coding of the data.
	Code literature and interview transcripts for evidence on hypotheses; Summarize findings by hypotheses	Code and summarize all interview transcripts and literature for evidence on hypotheses, specifically by looking for data on context-mechanism-outcome configurations
	Assess for quality and strength of evidence	Describe evidence according to strength (ie., whether the mechanism is supported by at least 2 sources of evidence) and triangulation of evidence (ie., whether the mechanism is supported by both interview and literature sources).
	Produce single case study report	Summarize case background, methods and findings into a single case report; undertake member checking by sharing findings with advisory group of policymakers
**Analyze data across cases**	Synthesize findings for each hypothesis across single case study reports to draw cross-case conclusions	Use results on support for hypotheses from single case studies to (i) categorize cases as theoretical replications or contrasts for each hypothesis and (ii) synthesize findings for each hypothesis across cases to draw cross-case conclusions; undertake member checking by sharing findings with advisory group
**Modify initial theory and hypotheses**	Modify initial theory and hypotheses	Use findings and compare to initial theory to make modifications based on cross-case findings

^1^ To minimize recall bias, cases should have engaged in the implementation of HiAP within the past seven years, but within 2–3 of initiation since it typically take time for implementation to develop after the initial mandate for HiAP.

^2^ Search strategy included in S1 File.

### Study hypotheses

According to Stake (2006) and Yin (2014), we can assess the strength of support for of specific pathways facilitating intersectoral engagement by examining the salience of multiple hypotheses across case studies [15,16]. Consistent with realist approaches, our study began with an initial theory (see [10] and set of initial hypotheses to be tested in Table 2). These hypotheses were generated based upon consultation with our advisory group of policymakers working in ISA and HiAP and informed by selected written documents and political theories [21,22]. These hypotheses are not meant to be mutually exclusive (ie., main and alternative); rather, we sought evidence to support the salience of each hypothesis individually with the expectation that they may all be useful in the implementation of HiAP.

**Table 2:** Initial hypotheses tested through our case study evidence.

**Hypothesis 1 –Awareness raising strategy**	In order for a government sector or non-governmental partner to engage in HiAP implementation, awareness of the need and reasons for an intersectoral approach to address health equity is sufficient.
**Hypothesis 2 –Directive strategy**	Beyond having an awareness of the need and reasons for an intersectoral approach to address health equity, a directive approach (ie., focused on health targets) is necessary to engage a government sector or non-governmental partner in HiAP implementation.
**Hypothesis 3 –Win-win strategy**	Beyond having an awareness of the need and reasons for an intersectoral approach to address health equity, a win-win approach (ie., focused on sector/partner-specific motivations and outcomes) is necessary to engage a government sector or non-governmental partner in HiAP implementation.

### Case selection

Cases were purposively selected from 16 HiAP initiatives that were implemented globally starting between 1980 and 2009, as identified and characterized in an earlier systematic scoping review [7]. This included implementation in diverse settings, including jurisdictions in Asia-Pacific, Europe, and North and South America. In selecting cases, our purpose was to encompass implementation occurring at different levels of jurisdictions (country, province, state) and to represent diverse mandates and governance structures for implementation in terms of legal and institutional background to HiAP. We were also aiming for comparability in the political and economic systems of the jurisdictions to help control for contextual differences found between our cases (Table 3). In particular, the mandate for HiAP was in the form of legislation in Sweden and Quebec; whereas implementation in South Australia was driven by a strategy. Also, Sweden is a nation-state; whereas South Australia and Quebec are sub-national jurisdictions.

**Table 3:** Contextual factors during the study period of the three case studies.

Contextual Factors	Sweden	Quebec	South Australia
**Mandate Type & Year**	Legislated in Swedish Public Health Objectives Bill, 2003	Legislated in Public Health Act, 2002	Strategy, 2008
**Mandate Description**	Minister of Health with directors-general of “concerned agencies” guides national-, regional- and local-level intersectoral health coordination with regards to the health policy.	All prospective policies that may impact population health must pass through a health impact assessment administered by the Ministère de la Santé et de Services sociaux (MSSS).	Health lens analyses are used to help government sectors meet targets laid out in the South Australia Strategic Plan (SASP) (2004, amendment in 2011)
**Structure**	Inter-ministerial committee guides national-, regional- and local-level intersectoral health coordination with regards to the health policy; Swedish National Institute of Public Health works with sectors to implement health impact assessments	Inter-ministerial committee whose purpose is raise awareness and educate to each Ministry about the implementation of Section 54; MSSS serves as advisory to ministry proposing policy; Institut national de santé publique du Québec works with sectors to implement health impact assessments	Chief Executives of the Department of the Premier and Cabinet engages governmental sectors to partner with health sector on SASP target; Health in All Policies Unit within the Health Department works with sectors to implement health lens analyses
**Level of Implementation**	Country, county, local	Province, region, municipality	State, local

### Key informant interviews

Verbal consent was obtained from participants given that interviews were always conducted by telephone. This specific procedure and the study was approved by the St. Michael's Hospital Research Ethics Board (#10–162).

To identify potential key informants, we began by reviewing all literature about the case to generate a preliminary understanding of HiAP implementation in each setting. We also used a snowball sampling strategy by asking key informants for referrals. In selecting key informants for interviews, we aimed for a diverse sample in terms of policy sectors and geographic levels of government, including some participants from outside of government. We often modified our search strategy as we completed more key informant interviews and identified new gaps in our understanding of HiAP implementation (eg., based on an initial review of the interview summary). We also screened for high self-rated familiarity with HiAP implementation.

All potential informants were screened for eligibility based on their self-rated familiarity with HiAP implementation on a Likert scale from very unfamiliar (1) to very familiar (5); those rating themselves as familiar (3) to very familiar (5) are deemed eligible. We intended to have between ten and 15 interviews per case, as we aimed to comprehensively investigate the diverse intersectoral activities that comprise implementation for each case (ie., within and across levels of government).

Telephone interviews with key informants were conducted in their native language using a semi-structured interview guide that focussed on hypotheses but that avoided leading key informants toward any one hypothesis and helped to rule out alternative explanations (Table 4). Questions aimed to understand the specific role of the informant in HiAP implementing, and to discuss examples of barriers and facilitators to implementation of HiAP across a range of themes relevant to our study hypotheses. Interviewers were instructed to probe for rich explanations for these phenomena (ie., answering ‘how’ and ‘why’ questions versus mere descriptions), including the relevance of contextual factors. Verbal consent was obtained from key informants given that interviews were always conducted by telephone.

**Table 4:** Number, job titles, and jurisdictions for key informants interviewed in Sweden, South Australia and Quebec.

Case	Number and job titles of key informants	Jurisdiction
**Sweden**	14: Politicians, public health experts, civil servants, academics, non-governmental organization employees	6 national, 8 local
**Quebec**	14: civil servants, public health experts, politicians	5 provincial, 9 regional/local
**South Australia**	14: civil servants, public health experts, sustainability planner, public intellectual	11 state, 3 regional/local

### Literature searching

We undertook a systematic search for evidence about our full set of case hypotheses. Literature was then analyzed in the same way as the interviews; that is, initial coding led to the summary of mechanisms (see below).

### Analysis

Like Pawson and Tilley (1997) [17], we sought to articulate mechanisms that explain how complex programmes work and fail in different contexts by developing context-mechanism-outcome (CMO) pattern configurations. For each of our three cases, two investigators independently coded interview data (using comments in Microsoft Word) and literature data (using comments in Adobe Acrobat) to flag text that directly referred to our hypotheses, focusing on rich description of mechanisms. All data was stored electronically in a system mirrored the capability of NVivo, ie., files were stored in clearly organized and labelled folders and filed in a shared drive that only the research team had access to.

Working as a team, we consolidated findings from each of the three case studies and undertook cross-case analysis to systematically synthesize data across cases for each hypothesis and to assess the strength of the evidence being summarized. In particular, to increase study rigor we used two types of triangulation in our approach [15,23]. First, we used multiple sources of evidence (ie., peer-reviewed and grey literature, and key informant interviews) to find support for our hypotheses. Second, we relied on multiple investigators to interpret evidence first independently and then in team meetings to discuss discrepancies. In order to ensure a transparent and consistent approach to conducting individual case studies, we conformed to a uniform case study protocol across cases for case selection, systematic literature searches, recruitment, interviewing, coding and analytic and cross-case synthesis processes [10,15].

## Results

Below we present summaries of our evidence including selected representative examples from interviews and the literature to illustrate supportive findings for our hypotheses.

### Awareness raising strategy

In all three cases, we found little evidence from either the literature or interviews that awareness raising activities motivated buy-in for HiAP by non-health sector partners by merely increasing the understanding of their role in implementation. Moreover, in the words of one informant, using this strategy alone led only to “small minor adjustments” to policies, i.e., weak buy-in.

In Sweden, for example, we found evidence at all levels of government that awareness raising was somewhat effective, but only when used in conjunction with other activities (eg., provision of financing for specific initiatives or using champions to motivate HiAP implementation). While our findings suggest that mere knowledge acquisition is not enough to motivate buy-in, we were not able to discern the relative importance of awareness raising when it was used more successfully in combination with other strategies.

Across case settings, we identified two main mechanisms for why awareness raising was not motivating buy-in for HiAP implementation among non-health sector partners. First, the objectives of projects related to the implementation of HiAP were not sufficiently in line with the core mission of some partners. Second, partners in other sectors were constrained by their desire to invest in projects that had a short timeline for realizing impacts; so, it was difficult to demonstrate the value of and justify action on HIAP implementation.

### Directive strategy

Evidence indicated that a directive strategy (in conjunction with or distinct from awareness raising) was sometimes counterproductive to obtaining buy-in for HiAP implementation. For instance, in Quebec, a public health bill mandated that health considerations been made during policy development across the government; and, yet, key informants at the provincial level noted that having a health-sector vision imposed on non-health sectors constrained their policy making process. Informants suggested that ISA was better facilitated through understanding the primary interests of non-health sectors. It appears that using a directive approach to facilitate agenda setting created tension about roles, responsibilities and accountability within non-health sectors in our case settings and may have ultimately slowed the policy making process.

### Win-win strategy

Ample evidence supported the hypothesis that a win-win strategy facilitated HiAP implementation. Informants described win-win strategies across all case settings, including at different levels of jurisdiction. In Sweden, emphasizing the "gains and rewards" of thinking beyond narrow perspectives of health and/or of doing more than “business as usual” led to buy-in from non-health sectors. A vision that implied a "win-win situation" or some "reciprocity" because it strengthened the "motivation for action” facilitated lasting HiAP engagement. According to Lawless (2012) [14], intersectoral engagement in South Australia sought to avoid being “health imperialists” (ie., a directive strategy) and to instead apply a “cooperation strategy”; thus, the health sector avoided defining the problem exclusively from a health perspective and responded to the needs and concerns of other sectors. Table 5 presents a summary of evidence regarding a range of win-win mechanisms that facilitated implementation by fostering agenda setting and capacity building.

**Table 5:** Availability of evidence by jurisdiction for each case on win-win mechanisms and the strength of support for each mechanism area.

Win-win mechanisms and resources	Evidence from jurisdictions			Support for evidence	
	Sweden	Quebec	South Australia	Strength^1^	Triangulation^2^
**Agenda setting**					
**Understanding mission and culture of other sectors and developing shared language**	Local	provincial, regional, local	state	strong	adequate
**Using dual outcomes to engage non-health sectors**	Local	-	state, local	strong	moderate
**Integrating health into the sustainability agenda**	national, local	-	-	strong	moderate
**Using scientific evidence to demonstrate effectiveness of HiAP**	Local	provincial	state	strong	adequate
**Policy coordination for public health outcomes to strengthen other policy proposals**	-	provincial	state	strong	adequate
**Using health impact assessment as a decision support tool**	Local	provincial	state, local	strong	adequate
**Capacity building**					
**Using community engagement strategies**	national, local	-	state, regional	strong	adequate
**Creating dedicated teams**	-	-	state	strong	moderate
**Using specific capacity building exercises**	regional, local	-	-	strong	poor
**Providing financial incentive to bring people to the table**	Local	local	local	strong	adequate
**Ensuring adequate time to develop shared language**	-	-	state, local	strong	adequate
**Maintaining engagement of high-level leadership**	-	-	state	strong	adequate

^1^ Strength of Evidence: The degree of support for the main/rival hypothesis across sources of evidence. Strong: Support is strong when there are at least 2 thick (rich and detailed descriptions) sources. Weak: Support is weak when there are fewer than 2 thick sources.

^2^ Triangulation: Evidence that is supported by multiple sources (ie., literature and interviews). Adequate: Strong evidence from both literature and interview. Low: Strong evidence from only one source (ie., literature or interviews). Poor: No strong evidence.

#### Agenda setting resources to facilitate perceived acceptability of HiAP

We learned how employing a win-win strategy using a range of specific resources facilitated agenda setting that improved the feasibility of implementing HiAP and thus, strengthened intersectoral engagement (Fig 1).

**Understanding sectoral missions and cultures to develop a shared language:** Evidence indicated that "every department views the problem under study first and foremost through the lens of its own mission" [24]. Thus, understanding the mission, goals and administrative culture of other sectors is essential for engaging them in a HiAP approach, through the development of a shared or common language and increased acceptability of HiAP.

In South Australia, an informant involved in managing HiAP activities highlighted the importance of recognizing different cultural practices for decision making, such as the high level of democratization in the education sector, and the infrequent use of consultations more characteristic of sectors strongly influenced by engineering, such as transportation. In Quebec, this cooperative approach was seen as effective at the municipal level when health sector actors adapted to other sectors’ "vocabulary, tools, network, issues and way(s) of doing things" [25]. In a similar way, in working on longer term collaborations for health, appealing to the short-term priorities of non-health sectors may lead to their long-term commitment. While discussing the implementation of local intersectoral collaborations under the Government Action Plan to Promote Healthy Lifestyles, one informant noted that by focusing on shorter-term goals with less emphasis on health equity and more directly on the “mission, concerns, funding issues” of partners can lead to longer-term awareness and appropriation of the shared benefits of collaboration.

**Using dual outcomes to demonstrate the value of HiAP for non-health sectors:** Informants at the state level in South Australia referred to the practice of framing policies in terms of “dual outcomes” as a strategy to facilitate non-health-sector buy-in. According to another informant, while working with partners on a health lens analysis (HLA) to improve family literacy and educational performance, it was refreshing for the health sector to work with them on their own agendas rather than vice versa (eg., focusing on education outcomes for vulnerable students rather than improving healthy eating behaviours). There was also evidence that this approach was fostering a more sustainable HiAP initiative. For example, another informant who worked on HiAP within the industry sector suggested that since South Australia first pioneered a collaborative approach to policy making, there had been an increasing appetite for multi-disciplinary/intersectoral approaches to policy. Thus, a good strategy for long-term HiAP seems to include other (non-health) outcomes into the process, in order to “morph” into a “broader connectiveness.”

**Integrating health into the agenda of other intersectoral strategies to rely on prior experience and utilize existing intersectoral structures and processes:** Another strategy that facilitates buy-in and HiAP implementation is to rely on intersectoral agendas already in place, because of the familiarity with social concepts and ‘automaticity’ in terms of intersectoral work [13]. An informant from the Swedish National Institute of Public Health (SNIPH) mentioned that rather than focusing on health, it was sometimes fruitful to use the concept of sustainable development as an entry point for intersectoral engagement, due to the Swedish government’s history of intersectoral collaboration regarding social sustainability. Another informant working on sustainable development initiatives within a municipal-level government used a variety of informal networks (ie., meetings with certain actors from diverse sectors) to raise awareness of the need for intersectoral action for health equity by making the links between health and other agendas (eg., sustainable development) more “visible” and by providing “inspiration for a different way of working”. On a practical level, these networks provided an opportunity for politicians to “step in and remind” participants about the need for health to be integrated into the common goals, and for various sectors to work out their respective responsibilities.

**Using scientific evidence to demonstrate effectiveness of HiAP and combat reluctance to invest in health:** We found strong evidence showing that using scientific evidence facilitated HiAP implementation both at the central and local levels by supporting economic arguments for HiAP acceptability. One informant in South Australia mentioned that, in the context of budgetary restraints at the state level, one successful win-win strategy for intersectoral engagement was to use evaluation evidence to demonstrate that HiAP can reduce the cost of curative services by preventing chronic disease burden through non-health sector actions. For partners within the health sector, this represented an opportunity to reduce costs, while for non-health sectors, actors recognized that expanding curative service costs are a threat to their own budgets. Thus, in engaging sectors in a HiAP approach, in part because of "current fiscal constraints", it is important to promote projects that are proven to be "cost-neutral" because they are more likely to buy-in. "When you put recommendations forward, about a particular responsibility a department has and it tweaks them or there’s some minor changes to that level or responsibility, some people’s perception is that, “Oh, this is going to cost us more money, therefore, no, we’re not going to do it.” […] Despite the fact that we know full well that it’s actually not going to cost them more; it’s simply just a slight change in their policy."

A limitation to this economic (cost-saving) argument is that considering the short political cycles and public interest in health care financing, it is challenging to raise awareness on the importance of ISA based on the argument that it is more cost-effective to invest in prevention than acting only on the level of curative interventions. However, objectives of sectors may in fact include 'maximization of societal and individual welfare', broader than simply cost saving, or financial objectives.

**Policy coordination for public health outcomes as a way of strengthening other policy proposals:** We found evidence at the highest levels of government that non-health sectors can be motivated to engage in HiAP implementation if participation strengthens the likelihood that those projects will be adoption. This is implicit in the approach taken with Sections 54 and 55 in Quebec, where the Minister of Health and Social Services has the power to delay policy adoption if health promotion is threatened. In South Australia, engaging in the HiAP process provides knowledge to non-health sectors about how their area impacts health, thereby supporting their own agenda, as it is "easier to sell the change or reform that they want by going through [HiAP processes]" [14]. In Quebec, one informant at the local level referred to the same mechanism in relation to an initiative led by the public transportation sector, where “the public health argument could support their project and helped them pass it”. In that sense, the win-win strategy facilitated acceptability of HiAP among non-health sectors by rewarding them for adopting a public health perspective.

**Using health impact assessment as a decision support tool to engage in HiAP:** Finally, we found that using health impact assessments in Sweden and Quebec and health lens analysis in South Australia was a motivator across all cases and jurisdictions, because these provide a "two-way dynamic" that “places equal emphasis on achieving the goals of other agencies at the same time as achieving health goals" [26]. At the local level in Sweden, using health impact assessment as a tool to aid and speed up decision making has been a key motivator for the implementation of HIAP in the Municipality of Nyashamn during construction of a highway bypass. Again, a motivation distinct from the simple awareness of the HiAP mandate was present: in this case, the municipality wanted a systematic analysis of health impacts; something an environmental impact assessment process did not include [27]. Another informant noted that the use of health lens analysis at the municipal level, in collaboration with the Department of Health in South Australia, was a useful tool to identify gaps in service provision and community needs.

#### Capacity building resources to facilitate perceived feasibility of HiAP

**Fig 2:**
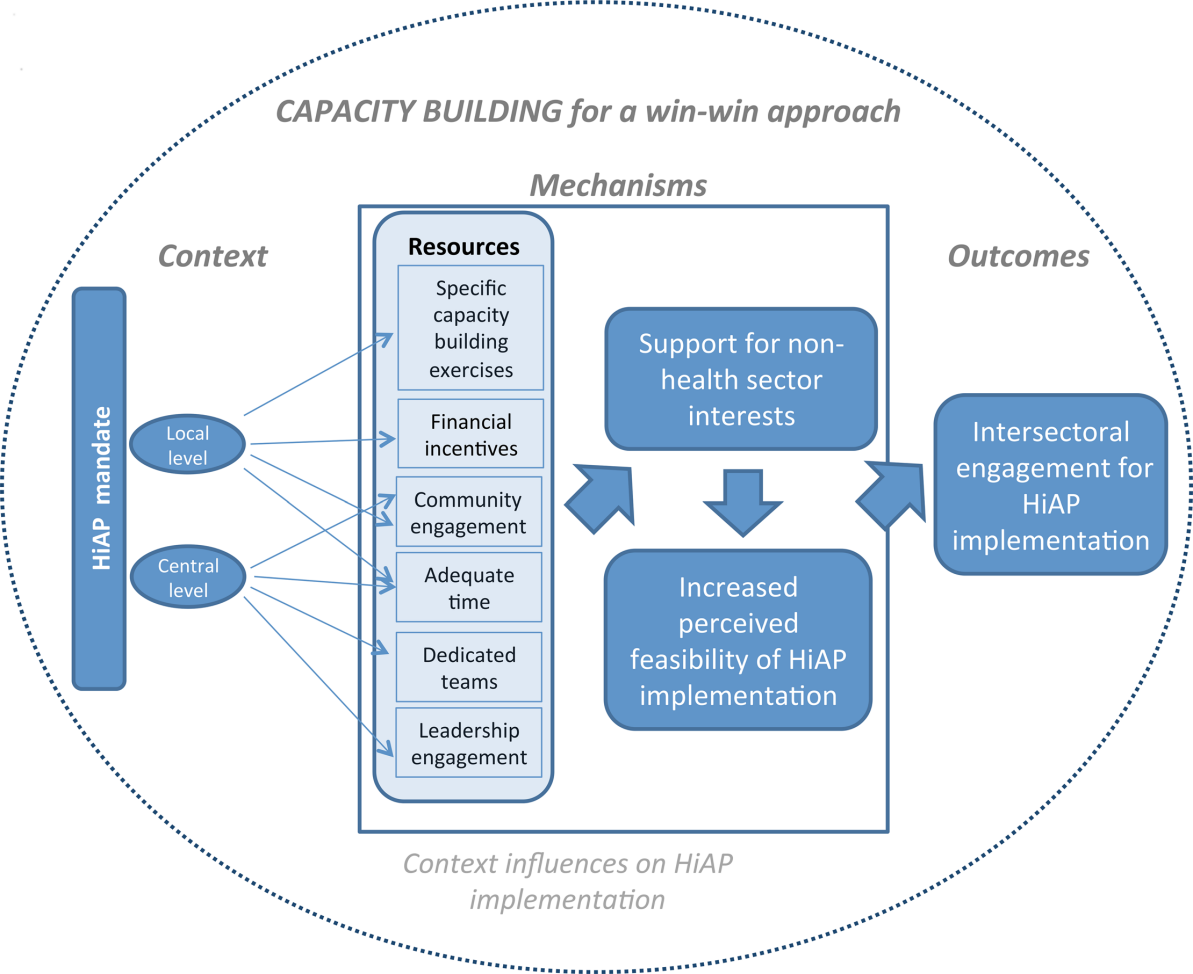
Refined Conceptual Framework–Agenda setting.

We also learned about how employing a win-win strategy using a range of specific resources facilitated capacity building that improved the feasibility of implementing HiAP and thus, strengthened intersectoral engagement (Fig 2).

**Using community engagement strategies to better understand and target stakeholder needs:** In South Australia, we learned about how continued collaboration on building healthy communities was strengthened by using specific communication strategies. One informant from the industrial sector in South Australia alluded to the “continuum of engagement”, noting that as a result of using a community engagement approach to policy development, communities became more receptive and HiAP was more feasible because a relationship had been built and they had become champions, as opposed to adjuncts to the decision-making process. In another example related to a regional development project, one informant recounted that especially when implementation requires “highly technical” solutions, an effort needs to be made to communicate information “in such a way that engages people”. In some cases, this may involve “using data as a hook to galvanize community thinking” and to encourage them to look at their problems from a different angle. Practitioners must, therefore, understand the most appropriate level of information to provide in order to “get the pitch right”.

**Creating dedicated teams to enhance and better coordinate knowledge and skills for HiAP engagement:** Several key informants in South Australia referred to the HiAP Unit (located within the Department of Health) as having the necessary skills to build trust and momentum around HiAP, and consequently increase feasibility of a concerted implementation approach. The success of a dedicated team relies on the expertise, freedom and legitimacy of team members, which enables them to act effectively across sectors (see also [28]).

**Use of specific capacity building exercises to develop supportive environment for the uptake of joined up approaches:** As a way to understand partners’ interests, some capacity building activities can stimulate longer-term partnerships. At the local level in Sweden, county administrative boards (i.e. the state's special regional representatives) worked with municipalities to develop locally relevant indicators and assignments. One informant identified these activities as important for increasing uptake of the Swedish Public Health Objective Bill and “incorporating public health into decision-making in other policy areas." Several informants in South Australia described memorandums of understanding (MoU) as useful tools for responding to the “needs of community” by building practical capacity and, in turn, putting a joined-up approach on the agenda at the local level.

**Providing financial incentive to bring people to the table and develop willingness for intersectoral work:** We found evidence across all cases that financial incentives can act as an effective facilitator for engagement in HiAP implementation at the local level. In Sweden, as municipalities are autonomous, county councils must create incentives to persuade municipalities to participate in intersectoral action. This raises the possibility that capacity building for HiAP strategies may be possible without an understanding of health inequalities or buy-in to the values of health equity, provided participating is beneficial and otherwise acceptable. In the context of implementing governmental health promotion initiatives through intersectoral collaboration at local level in Quebec, a key informant highlighted the importance of available funds to incentivize participation, which in the long-term can lead to continued collaboration and awareness of the shared benefits, even after financial incentives have lapsed. As an informant from South Australia noted, “it helps to have a little bit of money to be able to put on the table and leverage others so you can say well we’re putting this up there and you can get benefit out of it”.

**Ensuring adequate time for HiAP implementation to allow for developing a shared language:** We found evidence at all jurisdictional levels in South Australia showed the importance of leaving enough time to develop a win-win strategy. Project funding must allow sufficient time for discussion of "each other's business," as well as for systematically thinking through the health and well-being implications of policies and practices. As an informant at the regional level noted, when implementing programs with diverse partners at multiple levels, finding a “shared common ground and … synergies” is only feasible after spending time aligning conceptual understandings (e.g. nomenclature, language) that can bridge the sectoral cultural divides and allow to “delve deeper.” However, as one informant noted that in working to empower communities as a part of the HiAP strategy, senior managers were less likely to see the value of investing the required, significant amount of time and other resources (eg., in-kind support for human resources) in light of other pressures coming from their minister and their chief executives.

**Maintaining engagement of high-level leadership to ensure sustained mandate and resources for HiAP:** Sustainable implementation of HiAP requires continued support from the highest levels of government. In the context of implementing projects on the health targets of the South Australia's Strategic Plan focusing on "healthy weight," collaboration with partners from non-health sectors was based on an understanding of "where their policy leaders were", as well as looking at the impacts on health and well-being and how these benefited them. This approach made it possible to bring together a diverse group of highly committed partners and to develop the Eat Well Be Active Strategy. Another informant from South Australia suggested that ongoing support at the highest-levels of government can be only feasible by using “tailored messages… to continuously remind leaders about the importance of HiAP.” In turn, HiAP advocates needed to be capable of “navigating” leadership changes by finding ways for HiAP to “be able to still be relevant to the new cabinet priorities.”

## Discussion

Using a realist-informed explanatory multiple case study approach, our study provides some of the first evidence about how commonly used strategies for implementing HiAP *(ie*., *awareness raising*, *directive and win-win*) affect the use of HiAP ([29], see endnote). Through gathering and analyzing evidence to test our hypotheses, we were able to generate strong evidence and develop a refined understanding of how to foster HiAP implementation successfully.

**Fig 3:**
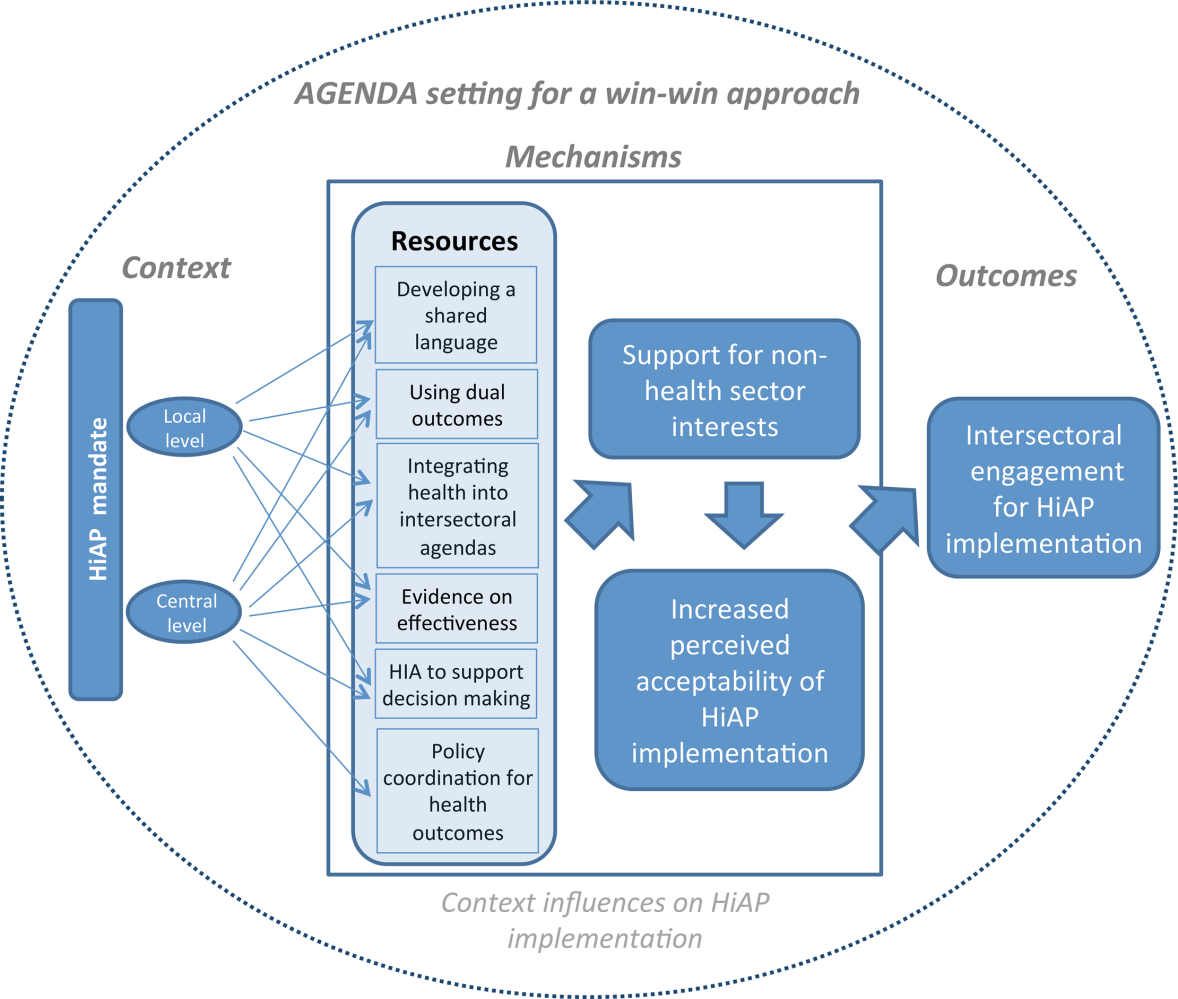
Refined Conceptual Framework–Capacity building.

We found the most abundant evidence that a win-win strategy emphasizing the relevance of outcomes for both health equity and specific goals of different partners was effective across all jurisdictions and cases. In particular, we found evidence on how and why the win-win strategy helped to foster both agenda setting and capacity building needed to obtain and maintain buy-in for HiAP implementation in different jurisdictions (as indicated in our refined conceptual frameworks presented in Figs 2 and 3, respectively).

Fafard (2013) has spoken of the challenges that governments interested in using HiAP face in maintaining so-called “integrated governance” [30]. Ollila (2011) described some possible strategies, including those we tested in this analysis [12]. Our findings about how and why different strategies for engaging policymakers into HiAP have and have not worked across our case settings helps health systems planners understand why they should consider the development of win-win partnerships with policy sectors a central part of their implementation plan. We also noted that different governance structures and tools were useful for supporting capacity and setting the agenda for HiAP when using this strategy.

In South Australia, greater emphasis was placed on building cross-sectoral relationships through understanding the goals of other sectors (win-win strategy) and letting a health focus emerge over time; although we found mechanisms related to this strategy in all case settings. South Australia also instituted a HiAP Unit that works to raise awareness, get buy-in for implementation and assist with health lens analyses, which has allowed for a more intensive approach to intersectoral engagement [26]. Other jurisdictions appeared to rely more heavily on awareness raising strategies. For example, the Swedish government used committees and informal meetings to raise awareness, and health impact assessments were conducted using the support of the SNIPH [31]; however there were apparently no civil servants that had the responsibility to engage with policy sectors to obtain buy-in for implementation. In Quebec, there was a mandate for health impact assessment embedded in the Province’s Public Health Act, and assessments were conducted using the support of the INSPQ; but again, there was not a strong role for intersectoral engagement.

On the other hand little evidence emerged to support the hypothesis that awareness raising alone is sufficient to engage sectors into HiAP. In Sweden, a variety of communication strategies were used (at all geographic levels of government) for the explicit purposes of raising awareness to facilitate agenda setting. However, evidence indicated that this strategy was not effective for agenda setting with all sectors, especially the business sector, as there was no apparent profit to be made from intersectoral work. Some evidence in Quebec and South Australia indicated that mere awareness raising is more effective at the local level, suggesting that less ‘political horse trading’ happens here; whereas at the highest levels of government, a win-win strategy is necessary to spur participation. However, it should be noted that due to the continuous and targeted awareness raising activities before the adoption of the South Australia Strategic Plan (i.e. in phase of initiation), there was already an “awareness” and “commitment to social policy” in place within the South Australia government that recognized the value of HiAP.

Similarly, we found no strong support for use of a directive approach to facilitate HiAP implementation across cases. We expected more support for this strategy in Quebec, based on the legally mandated use of health impact assessment laid down in Section 54 of the Public Health Act. However, several informants in Quebec referred to the importance of a win-win strategy, saying “less preaching and more serving” was needed to generate interest and buy-in for HiAP amongst non-health sectors. A potential explanation for this finding is that although Section 54 represents social consensus regarding "collective responsibility for population health", and acts as a significant lever for action, it is still not a sufficient incentive for engaging non-health sectors in HiAP implementation [25,31].

While some win-win mechanisms were observed in all case settings, we also found others to be idiosyncratic to certain case settings (S1 Table). Sweden was the only setting where we observed some support for all of the win-win mechanisms for agenda setting; in particular, at the local level. The win-win strategy was likely of high importance at this level because of the relatively strong autonomy of county and municipal-level authorities. That all strategies were somewhat effective in Sweden may partly reflect the unique political context (eg., traditionally strong social democratic values and a strong welfare state [31,32]), which could have led to a political culture that facilitated HiAP implementation there. In South Australia, we found support for almost all of the agenda setting and capacity building mechanisms related to the win-win strategy at the state level (i.e., central government), which may reflect the central role of the HiAP Unit, which was staffed by a full-time workforce to engage stakeholders from non-health sectors to conduct health lens analyses.

Some mechanisms were only supported by evidence from a specific level of government; others were found at different levels. For example, policy coordination for public health outcomes appeared only at the highest level of government in Quebec and South Australia as a successful practice to stimulate the decision making process. Financial incentives, meanwhile, are of more importance at the local level in all cases to facilitating intersectoral collaboration, and build capacity by bringing people to the table. This highlights the importance of considering the local context of implementation (ie., within each jurisdiction of government) when developing a strategy for implementation HiAP.

We found that a variety of different types of resources could be used to facilitate a win-win strategy, including cultural (eg., developing a shared language), economic (eg., financial incentives) and political (eg., community and leadership engagement, using dual outcomes) resources. Moreover, we found that certain resources were more or less utilized for win-win strategies in different case settings. We have previously argued that a systems view of HiAP implementation would be useful for explaining how this process occurs in different contexts [4], and others have also espoused systems theory for explaining implementation [33–35]. More attention should be paid to developing systems theory specific to HiAP implementation to improve explanation of implementation outcomes.

While our study focused on testing hypotheses related to three strategies for intersectoral engagement, there are other strategies that have been described more recently and that are not directly examined in our analysis. For example, Katikireddi et al (2014) found evidence from Scotland that the framing of public health issues affected how much of a priority these issues had in setting the policy agenda among public, voluntary and industry stakeholders [36]. Our finding that the win-win strategy was particularly useful for getting buy-in from multisectoral policymakers is not inconsistent with this finding given that it can be viewed as a form of policy framing.

One limitation of our study was that our sample was comprised of three cases set in relatively similar contexts (eg., high-income democracies), which could limit the generalizability of our findings. For example, a HiAP is also utilized in Thailand and Iran, both of which are middle income countries and have distinct political traditions [37]; so it is unclear how necessary or effective a win-win approach would be in those settings. As well, though we endeavoured to probe for alternative explanations for phenomena during key informant interviews, they may not have revealed important mechanisms, because we could not always recruit key informants from all sectors involved at all levels of geography within a case, and also individuals are limited by their own perspective (notably influenced by their position, eg., manager, researcher, politician) on the barriers and facilitators that they experienced. More fundamentally, it is worth noting that our analysis focused on learning about strategies for improving the implementation of HiAP in terms of intersectoral engagement, and not with respect to the impact that HiAP can have on improving population health and equity.

Nevertheless, a key strength of our study was that it supported theory building and learning about causal linkages between the inner mechanisms of HiAP functioning, combining multiple case study and realist approaches. We aimed to gather a comprehensive view of implementation phenomena across the interviews conducted and took several measures to address this objective. Key informants were recruited discreetly and guaranteed anonymity; both of which would minimize the risk that key informants would feel like they had to be partisan in their explanation of implementation outcomes. Key informants also had different levels of engagement with the government, including some key informants from outside of the government, which increased the opportunity to hear about HiAP implement from diverse stakeholder perspectives. During interviews, when key informants told us about an aspect of implementation that they were unable to clearly explain, we often asked key informants to refer us to other key informants who might be more knowledgeable (an example of our snowball sampling approach). Finally, our semi-structured interview guide included questions and probes to gather detailed explanations of implementation outcomes and to rule out alternative explanations (ie., explanatory versus descriptive case studies).

The mechanisms and successful practices uncovered by our results may enable actors in a diversity of settings to create positive conditions for implementing HiAP by supporting collaborative processes and outcomes across sectors, both within and outside of government, through improving the skills and capacities of individual actors taking a leadership role in a variety of initiatives.

## Supporting Information

S1 File Example of search strategy for Swedish case study. (DOCX)

S1 Table Examples for context-outcome-pattern configurations. (DOCX)
