# Supplementary material for: Using Win-Win Strategies to Implement Health in All Policies: A Cross-Case Analysis
Source: PLoS One. 2016 Feb 4;11(2):e0147003. doi: 10.1371/journal.pone.0147003 (PMC4742077; doi:10.1371/journal.pone.0147003)
Supplement: S1 File — (DOCX) [file pone.0147003.s001.docx]

**S1 File. Example of search strategy for Swedish case study**

***Search terms to be applied***

We used a combination of generic terms (eg., Health in All Policies) with other terms that relate to the name of specific HiAP policies, including a piece of formal legislation, a strategy, or some cross-sectoral committee or council that is central to the HiAP approach in a setting. For example, in Sweden, HiAP is represented by the National Public Health Objectives.

- “Health in all policies” AND Sweden
- “Health equity in all policies” AND Sweden
- “health on equal terms” w/ “Sweden”
- “Public Health Objectives” *or* “national objectives for public health” w/ “Sweden”
- “Public Health Committee” w/ “Sweden”
- “Intersectoral-” w/ “policy *or* collaboration *or* action *or* cooperation” w/ “health *or* equity *or* ineuquity” AND Sweden
- “Interministerial” w/ “policy *or* collaboration *or* action *or* cooperation” w/ “health *or* equity *or* ineuquity” AND Sweden
- “Intergovernmental” w/ “policy *or* collaboration *or* action *or* cooperation” w/ “health *or* equity *or* ineuquity” AND Sweden
- “Horizontal” w/ “policy *or* collaboration *or* action *or* cooperation” w/ “health *or* equity *or* ineuquity” AND Sweden
- “Intersect*or*ality” w/ “health *or* equity *or* ineuquity” AND Sweden
- “Horizontal government” w/ “health” AND Sweden
- “Joined up government” *or* “Joined up policy” w/ “health” AND Sweden
- “Healthy public policy” AND Sweden
- “Policy coordination” *or* “Coordinated policy” w/ “health” AND Sweden
- “Social determinants of health” w/ “policy” AND Sweden
- “Health for all” AND Sweden
- “Health impact assessment” w/ “policy” AND Sweden
- “Health equity impact assessment” w/ “policy” AND Sweden
- “Health equity” w/ “policy *or* collab*or*ation *or* action *or* cooperation” AND Sweden
- “Whole of Government” w/ Health AND Sweden

***Academic databases to search for literature***

- PubMed
- Medline
- CINAHL
- EMBASE
- PAIS International (Public Affairs Information Service)
- Worldwide Political Science Abstracts
- International Bibliography of the Social Sciences Applied Social Sciences Index Abstracts (ASSIA)
- ProQuest Political Science
- Left Index

***Journals to hand search***

These journals do not appear in the selected databases. The above search terms were applied to search each journal.

- Health Policy (Amsterdam, Netherlands)
- Health Policy Quarterly
- Health Policy Week
- The Journal of American Health Policy
- BMC Health Research Policy and Systems
- Journal of Public Policy
- Canadian Public Policy
- Journal of European Public Policy

***Internet search for grey literature***

- Key in search terms (as described above) in Google
- The first **30 hits** per search term will be ‘reviewed for relevant information, documents, and other links of relevance to HiAP activities described the original case summary.

***Review reference lists of relevant articles***

- Peruse the work cited list of articles that have been particularly useful, for potentially relevant articles.
- Download articles not already in database
