# Supplementary material for: Using Win-Win Strategies to Implement Health in All Policies: A Cross-Case Analysis
Source: PLoS One. 2016 Feb 4;11(2):e0147003. doi: 10.1371/journal.pone.0147003 (PMC4742077; doi:10.1371/journal.pone.0147003)
Supplement: S1 Table — (DOCX) [file pone.0147003.s002.docx]

**S1 Table. Examples for context-outcome-pattern configurations**

| **Win-win mechanisms** | **Context-outcome-pattern (CMO) configuration** | **Evidence from jurisdictions** |
| --- | --- | --- |
|  | **CMO examples supported by a single case** |  |
| Integrating health into the sustainability agenda | In the context of implementing Healthy Cities – a network that works towards common goals with similar strategies in addressing the social determinants of health – a municipal level informant from the sustainable development sector indicated that agenda setting was facilitated through local networks by making the links between health and other agendas (i.e., sustainable development) more “visible”, reminding various sectors about the need for health to be integrated into the common goals, and ultimately providing “inspiration for a different way of working”. | Sweden, national and local levels |
| Creating dedicated teams | When working with other sectors, intersectoral collaboration can be facilitated by expert teams with the freedom and legitimacy to not focus explicitly on being “advocates” for health in cross-sectoral collaborations, such as in case of the South Australian HiAP Unit. | South Australia, state level |
|  | **CMO examples supported by multiple cases** |  |
| Using dual outcomes to engage non-health sectors | Intersectoral collaboration can be facilitated by framing discussions to strengthen determinants of health in terms of policy priorities outside of the traditional health sector. E.g., when working with agricultural sector, rather than talking about obesity, contaminated food or improving nutrition, must learn how to frame in terms of whole government food policy, including farming and agricultural development and local markets. | Sweden local level;  South Australia state and local level |
| Understanding mission and culture of other sectors and developing shared language | In Quebec, the implementation of the Government Action Plan to Promote Healthy Lifestyles was best served when focusing directly on the “mission, concerns, funding issues” of partner. The end result was that there was longer-term awareness and appropriation of the shared benefits of collaboration (e.g., improving child outcomes in the community). | All cases, all levels |
| Using scientific evidence to demonstrate effectiveness of HiAP | At the provincial level in Quebec, an informant working in the Ministry of Public Health stated that using a health equity argument alone may lead sectors to “tell us that they hear us, but, [they may] not address it in the way we would like them to”. In particular, economic evaluation was mentioned as an important tool to engage non-health sectors in HiAP implementation, as it allows produces knowledge the benefits of HiAP which can be communicated to non-health sectors. Economic arguments carry more weight with some non-health sectors. | All cases, all levels |
